# Prognostic Nutritional Index (PNI) in Patients With Diabetes and Sepsis: A Cross-Sectional Study

**DOI:** 10.7759/cureus.104004

**Published:** 2026-02-20

**Authors:** Shubhransu Patro, Sidharth S Pattnaik, Parmarth Arora, Arushi Choudhary, Vibha Sharma, Prithviraj U Naik, Mayank Arora, Simoni B Pandit

**Affiliations:** 1 General Medicine, Kalinga Institute of Medical Sciences, Bhubaneswar, IND

**Keywords:** correlation analysis, healthcare associated infection, immune-inflammation indexes, low serum albumin, lymphocyte count, multi-organ dysfunction syndrome (mods), prognostic nutritional index (pni), sequential organ failure assessment (sofa), severe sepsis, vasopressor

## Abstract

Background and objectives: Sepsis and multiorgan dysfunction syndrome (MODS) are serious global health concerns. Patients with critical illnesses are assessed using the sequential organ failure assessment (SOFA) score for morbidity. The prognostic nutritional index (PNI) is a novel composite biomarker for sepsis. It is based on two parameters: absolute lymphocyte count and serum albumin. The purpose of this study was to examine PNI in patients with diabetes and sepsis. Additionally, we correlated the SOFA score and PNI of the study participants at admission.

Methods: This cross-sectional study was carried out from August 2025 to December 2025 at the Kalinga Institute of Medical Sciences (KIMS), Bhubaneswar, India. Adult diabetes patients of both sexes fulfilling Sepsis-3 criteria were included in this study. We recorded their absolute lymphocyte count and serum albumin for PNI calculation. SOFA scores were noted at day 1, 3, and 7. The categorical and continuous variables were assessed with the Chi-square and the Wilcoxon test, respectively. We used the Spearman correlation to assess the association between the SOFA score at day 1 and the subjects' PNI values. R software (version 4.3.2) was used for data analysis.

Results: A total of 557 patients were assessed in this study. Their median age was 59.0 (52.0-65.0) years. Of the participants, 335 (60.14%) were male. The average hospital stay was 12.0 (9.0-16.0) days. MODS was recorded in 459 (82.41%) patients. The median serum albumin and absolute lymphocyte count of the study population were 2.80 (2.40-3.20) g/dL and 1.13 (0.69-1.88) x 10^9^/L, respectively. The median PNI of the subjects was 34.64 (interquartile range (IQR), 30.32-39.39). The median SOFA score during admission was 5.0 (IQR, 3.0-8.0). There was a negative correlation between SOFA score at day 1 and PNI (-0.153, 95% CI: -0.233 to -0.071, p < 0.001). Male and female subjects demonstrated similar negative correlations.

Conclusion: Male and female subjects with sepsis had comparable PNI values. However, serum albumin, absolute lymphocyte count, and SOFA scores showed statistically significant differences between genders. The PNI and SOFA scores were negatively correlated. The majority of the subgroup analyses also showed weakly negative associations. However, the study findings cannot be generalized due to a single-centric design, a small sample size, and missing data on hemodynamic, renal, hepatic, and glycemic parameters, antibiotics used, daily fluid intake, urine output, comorbidities, and concomitant medications.

## Introduction

Sepsis is characterized as a potentially fatal organ failure caused by an infection-related dysregulated host response [1,2]. The death rates from sepsis and severe sepsis were reported to be 17% and 26%, respectively, in a recent meta-analysis comprising 15 studies [3]. The prevalence of diabetes is increasing worldwide due to dietary and lifestyle changes [4]. The risk of infection and sepsis is 20% higher in individuals with diabetes than in those without diabetes [2,5,6].

Multiorgan dysfunction syndrome (MODS) is the final stage of sepsis. According to recent studies, the primary pathophysiological mechanism of sepsis is immunological dysfunction triggered by dysregulated systemic inflammatory response [7-10]. Inflammatory mediators, immunological dysfunction, gut microbiota imbalance, and gene polymorphisms are all strongly linked to sepsis and MODS in diabetic individuals [8,11-13].

The Sequential Organ Failure Assessment (SOFA) score was developed to assess the severity of illness in critically ill patients [14]. C-reactive protein (CRP), procalcitonin, interferons, interleukin-6 (IL-6), adrenomedullin, and intercellular adhesion molecule-1 (ICAM-1) are newer biomarkers for sepsis [15,16]. Higher cost and limited availability render these biomarkers as less feasible alternatives for quantifying sepsis progression [15,17].

The prognostic nutritional index (PNI) is a new composite biomarker for sepsis. It is based on two parameters, i.e., serum albumin and absolute lymphocyte count. PNI is calculated by the following formula: serum albumin (g/L) + 5 × lymphocyte count (10^9^/L) [18-20]. It is used as a biomarker of sepsis in patients with acute kidney injury (AKI), thromboembolic events, and acute respiratory distress syndrome (ARDS) [18,21]. A higher risk of hemodynamic instability and organ dysfunction may be reflected by a lower PNI value at admission [18-21]. In critically ill sepsis patients, the PNI shows an inverse relation with SOFA scores, indicating its potential as an early risk classification tool [22]. Hence, this study was conducted to evaluate and compare the PNI of patients with sepsis. We also correlated the PNI and SOFA scores of the participants during admission.

## Materials and methods

This cross-sectional study was conducted from August 4, 2025, to December 29, 2025, at the Kalinga Institute of Medical Sciences (KIMS) in Bhubaneswar, India. The study was approved by the Institutional Ethics Committee KIMS (approval number: KIIT/KIMS/IEC/2270/2025, dated 27.07.2025).

Eligibility criteria

Adult patients of both sexes with diabetes admitted to our hospital during the study period who met Sepsis-3 criteria [23] were included. Patients with any malignancy, immunocompromised state, ongoing chemotherapy, steroids, or blood component transfusion were excluded. We also excluded patients referred from other hospitals and those who died within or stayed for less than seven days.

Study procedure

Sociodemographic data (i.e., age, sex, socioeconomic status, and marital status) of the patients were collected from their discharge summaries. The Kuppuswamy classification was used to classify the socioeconomic class [24]. The following clinical parameters were noted at admission to the hospital: serum albumin and absolute lymphocyte count. The SOFA scores were assessed on the day of admission, day 3, and day 7, as per the SOFA scoring system [14]. The differences in the SOFA scores of the study participants were calculated at days 1 and 7. The PNI was computed from the collected values for serum albumin and absolute lymphocyte count. The normal ranges of serum albumin and lymphocyte count are 3.5-5.0 g/dL and 1.0-4.8 x 10^9^ cells/L, respectively. The correlation between baseline SOFA score and PNI of the patients was assessed, and a subgroup analysis of the correlation based on the outcome (i.e., death or discharge), difference in SOFA score was done at day 7 (<2 or ≥2), vasopressor (required or not), MODS (present or not), age group (≤65 or >65 years), and duration of hospitalization (>14 or ≤14 days).

Statistical analysis

For this study, a non-probability consecutive sampling was used. The Shapiro-Wilk test was used to assess the normality of data distribution. The continuous variables were reported as medians and interquartile ranges (IQRs). Categorical variables were presented as numbers and percentages. The categorical and continuous variables were analyzed with the Chi-square and the Wilcoxon test, respectively. The test statistics for the categorical and continuous variables were Chi-square value and t-value, respectively. We used the Spearman correlation to assess the association between baseline SOFA scores and PNI in the subjects. We reported the correlation coefficients with 95% confidence intervals (CIs). For data analysis, R software version 4.3.2 (R Foundation for Statistical Computing, Vienna, Austria) [25] was used. A p-value ≤ 0.05 was considered statistically significant.

## Results

During the study period, 2194 patients were admitted to the medicine ward. Of these, 904 patients were non-diabetic, 494 patients either died or left the hospital against medical advice within seven days of their admission, and 239 did not develop sepsis. The data from the remaining 557 patients were analyzed in this study.

Table 1 shows the clinical and sociodemographic data of the 557 patients included in the study. The median age of the study population was 59.0 (52.0-65.0) years. The majority of the participants were male (n=335, 60.14%). The median duration of hospitalization was 12.0 (9.0-16.0) days. A total of 175 (31.42%) patients were hospitalized for > 14 days. MODS was recorded for 459 (82.41%) patients. Vasopressors were required for 306 (54.94%) patients. The median SOFA score at admission was 5.0 (3.0-8.0).

**Table 1:** Demographic and clinical traits of the study population The categorical variables were presented as numbers and percentages, and assessed with the Chi-square test. The continuous variables were presented as median and IQR, and assessed with the Wilcoxon test. The test statistics for categorical and continuous variables were the Chi-square value and the t-value, respectively. The statistical significance is set at p < 0.05. The SOFA scores were assessed on the day of admission, day 3, and day 7, as per the SOFA scoring system [14]. The normal ranges of albumin and absolute lymphocyte count are 3.5-5.0 g/dL and 1.0-4.8 x 10^9^ cells/L, respectively. IQR: interquartile range, PNI: prognostic nutritional index, SOFA: sequential organ failure assessment score, MODS: multiorgan dysfunction syndrome

Parameters	Total (n = 557)	Male (n = 335)	Female (n = 222)	Statistical test used	Test statistics	p-value
Age (years), median (IQR)	59.00 (52.00-65.00)	60.00 (53.00-66.00)	58.00 (49.25-64.00)	Wilcoxon test	4.064	0.022
Elderly (Age > 65 years), n (%)	125 (22.44%)	88 (26.27%)	37 (16.67%)	Chi-square test	171.939	< 0.001
Marital status, n ($)						
Married	502 (90.13%)	300 (89.55%)	202 (91.00%)	Chi-square test	152.781	< 0.001
Unmarried	34 (6.10%)	23 (6.87%)	11 (4.95%)			
Divorced/widowed	21 (3.77%)	12 (3.58%)	9 (4.05%)			
Socioeconomic status, n (%)						
Low	335 (60.14%)	217 (64.78%)	118 (53.15%)	Chi-square test	191.236	< 0.001
Lower middle	198 (35.55%)	101 (30.15%)	97 (43.70%)			
Upper middle	24 (4.31%)	17 (5.07%)	7 (3.15%)			
Serum albumin (g/dL), median (IQR)	2.80 (2.40-3.20)	2.90 (2.50-3.30)	2.70 (2.30-3.12)	Wilcoxon test	9.670	0.003
Absolute lymphocyte count (10^9^/L), median (IQR)	1.13 (0.69-1.88)	1.06 (0.65-1.73)	1.26 (0.78-2.12)	Wilcoxon test	3.926	0.025
PNI, median (IQR)	34.64 (30.32-39.39)	34.74 (30.76-39.56)	34.26 (29.50-39.38)	Wilcoxon test	0.152	0.170
Hospitalization (days), median (IQR)	12.0 (9.0-16.0)	12.0 (10.0-17.0)	12.0 (9.0-14.0)	Wilcoxon test	0.073	0.589
SOFA score at day 1, median (IQR)	5.0 (3.0-8.0)	5.0 (3.0-9.0)	4.0 (2.0-8.0)	Wilcoxon test	3.104	0.041
SOFA score at day 3, median (IQR)	6.0 (3.0-9.0)	6.0 (3.0-9.0)	6.0 (2.3-9.0)	Wilcoxon test	0.064	0.671
SOFA score at day 7, median (IQR)	6.0 (2.0-10.0)	6.0 (2.0-10.0)	5.0 (2.0-9.0)	Wilcoxon test	0.193	0.137
Difference in SOFA scores, median (IQR)	0.0 (-2.0-2.0)	0.0 (-2.0-2.0)	0.0 (-2.0-3.0)	Wilcoxon test	0.086	0.491
MODS, n (%)	459 (82.41%)	285 (85.07%)	174 (78.38%)	Chi-square test	25.174	< 0.001
Vasopressor required, n (%)	306 (54.94%)	177 (52.84%)	129 (58.11%)	Chi-square test	17.839	< 0.001
Long duration of stay (hospitalization > 14 days), n (%)	175 (31.42%)	125 (37.31%)	50 (22.52%)	Chi-square test	144.616	< 0.001

**Figure 1:**
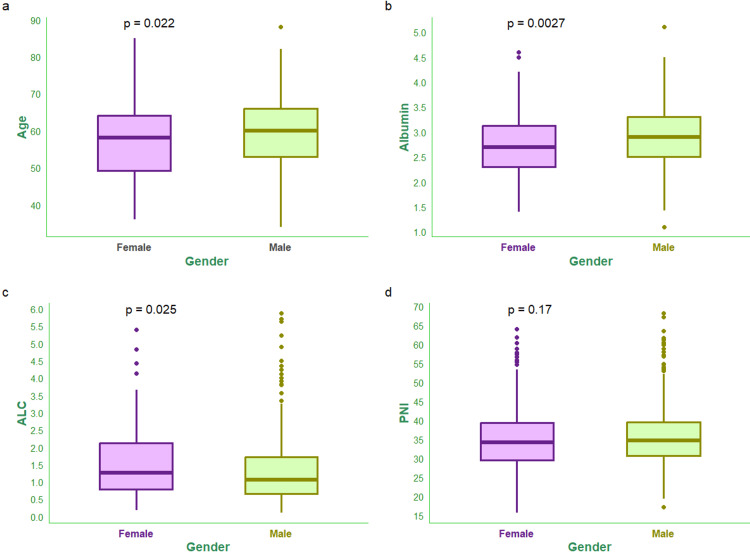
Age and PNI of the study population The box-and-whisker plots show age and PNI (along with its components, i.e., albumin and lymphocyte count) for female and male participants. Panels a, b, c, and d show the age (years), serum albumin (g/dL), absolute lymphocyte count (x 10^9^/L), and PNI, respectively. The statistical significance is set at p < 0.05. The intergroup comparisons were performed with the Wilcoxon test. ALC: absolute lymphocyte count, PNI: prognostic nutritional index

Figure 1 shows age, PNI, and its components (i.e., serum albumin and absolute lymphocyte count) of the study population. The median ages of male and female subjects were 60.00 (IQR, 53.00-66.00) years and 58.00 (IQR, 49.25-64.00) years, respectively (Figure 1a). The median serum albumin values of male and female subjects were 2.90 (IQR, 2.50-3.30) g/dL and 2.70 (IQR, 2.30-3.12) g/dL, respectively (Figure 1b). The median absolute lymphocyte counts of male and female subjects were 1.06 (IQR, 0.65-1.73) x 10^9^/L and 1.26 (IQR, 0.78-2.12) x 10^9^/L, respectively (Figure 1c). The median PNI values of male and female subjects were 34.74 (IQR, 30.76-39.56) and 34.26 (IQR, 29.50-39.38), respectively (Figure 1d).

**Figure 2:**
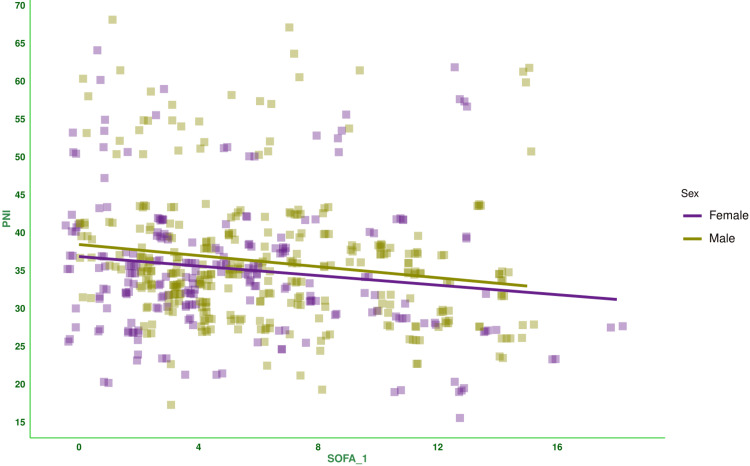
Correlation between PNI and SOFA score of female and male participants The jitter plots show the correlation between baseline SOFA score and PNI of the study population. The Spearman correlation was used to check the association. The SOFA scores were assessed on the day of admission, day 3, and day 7, as per the SOFA scoring system [14]. The statistical significance is set at p < 0.05. PNI: prognostic nutritional index, SOFA: sequential organ failure assessment score, SOFA_1: SOFA score at day 1

**Figure 3:**
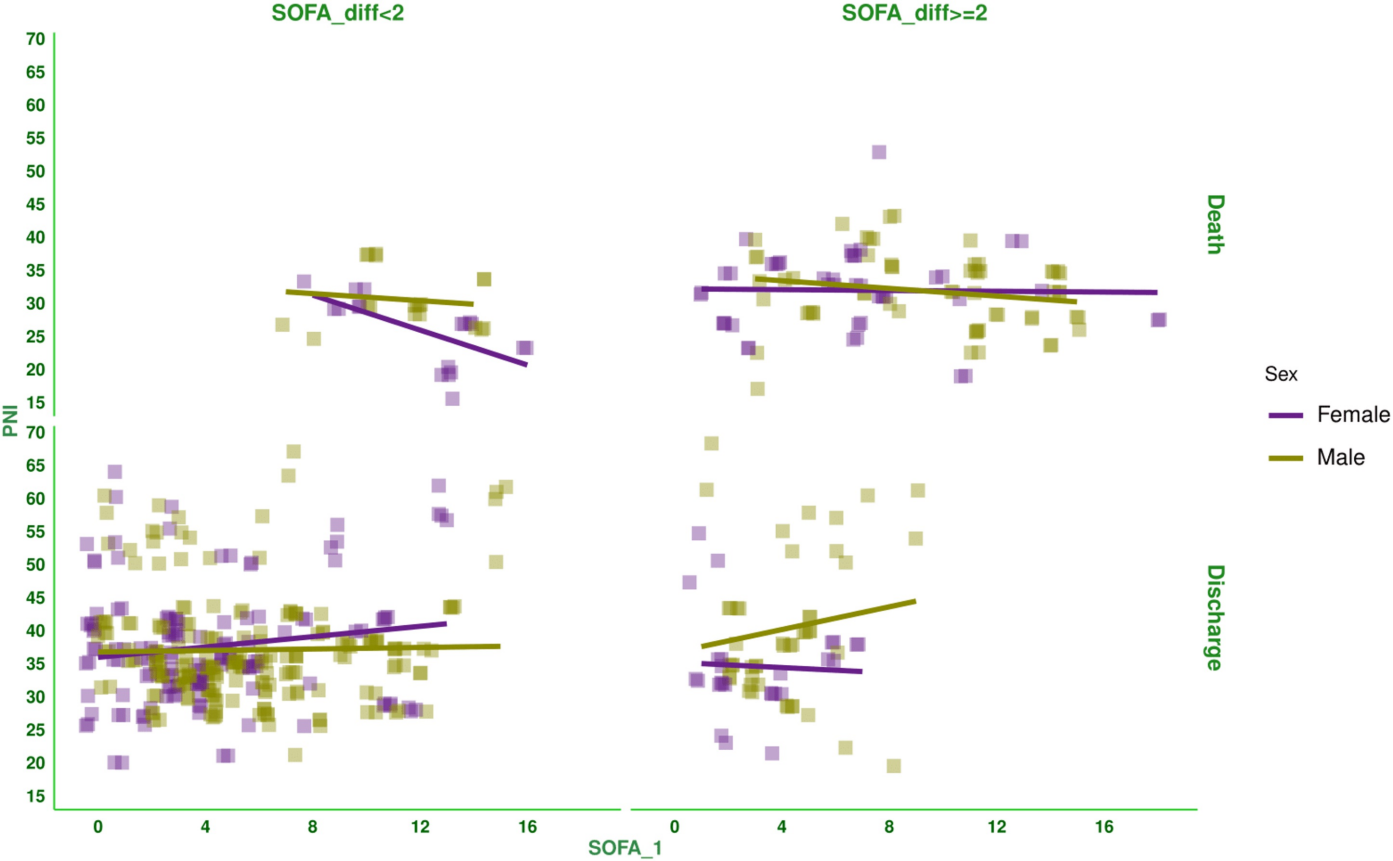
Correlation between PNI and SOFA score based on outcome and the difference in SOFA scores of the study population The jitter plots show the correlation between baseline SOFA score and PNI of the study population. The horizontal and vertical grids present the outcome (i.e., death or discharge) and difference in SOFA score at day 7 from that at day 1 (<2 or ≥2), respectively. The Spearman correlation was used to analyze the association. The SOFA scores were assessed on the day of admission, day 3, and day 7, as per the SOFA scoring system [14]. The statistical significance is set at p < 0.05. PNI: prognostic nutritional index, SOFA: sequential organ failure assessment score, SOFA_1: SOFA score at day 1, SOFA_diff: difference in SOFA score at day 7 from that at day 1

**Figure 4:**
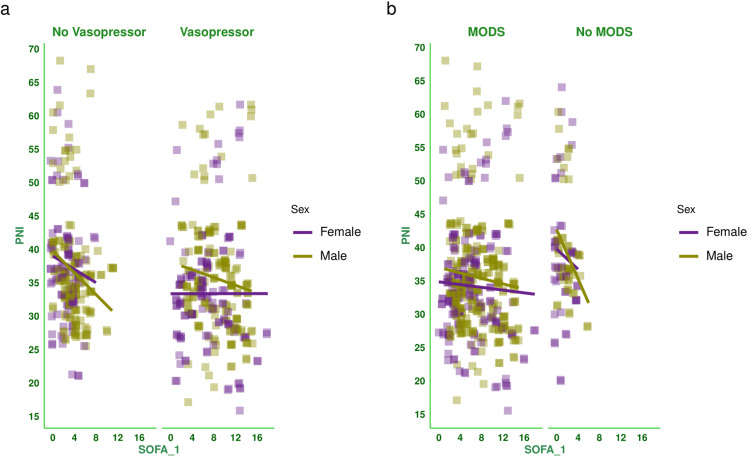
Correlation between PNI and SOFA score based on the requirement of vasopressors and presence of MODS The jitter plots show the correlation between baseline SOFA score and PNI of the study population. Panels a and b show the subgroup analyses based on the requirement for vasopressors and the presence of MODS, respectively. The Spearman correlation was used to check the association. The SOFA scores were assessed on the day of admission, day 3, and day 7, as per the SOFA scoring system [14]. The statistical significance is set at p < 0.05. PNI: prognostic nutritional index, SOFA: sequential organ failure assessment score, SOFA_1: SOFA score at day 1, MODS: multiorgan dysfunction syndrome

**Figure 5:**
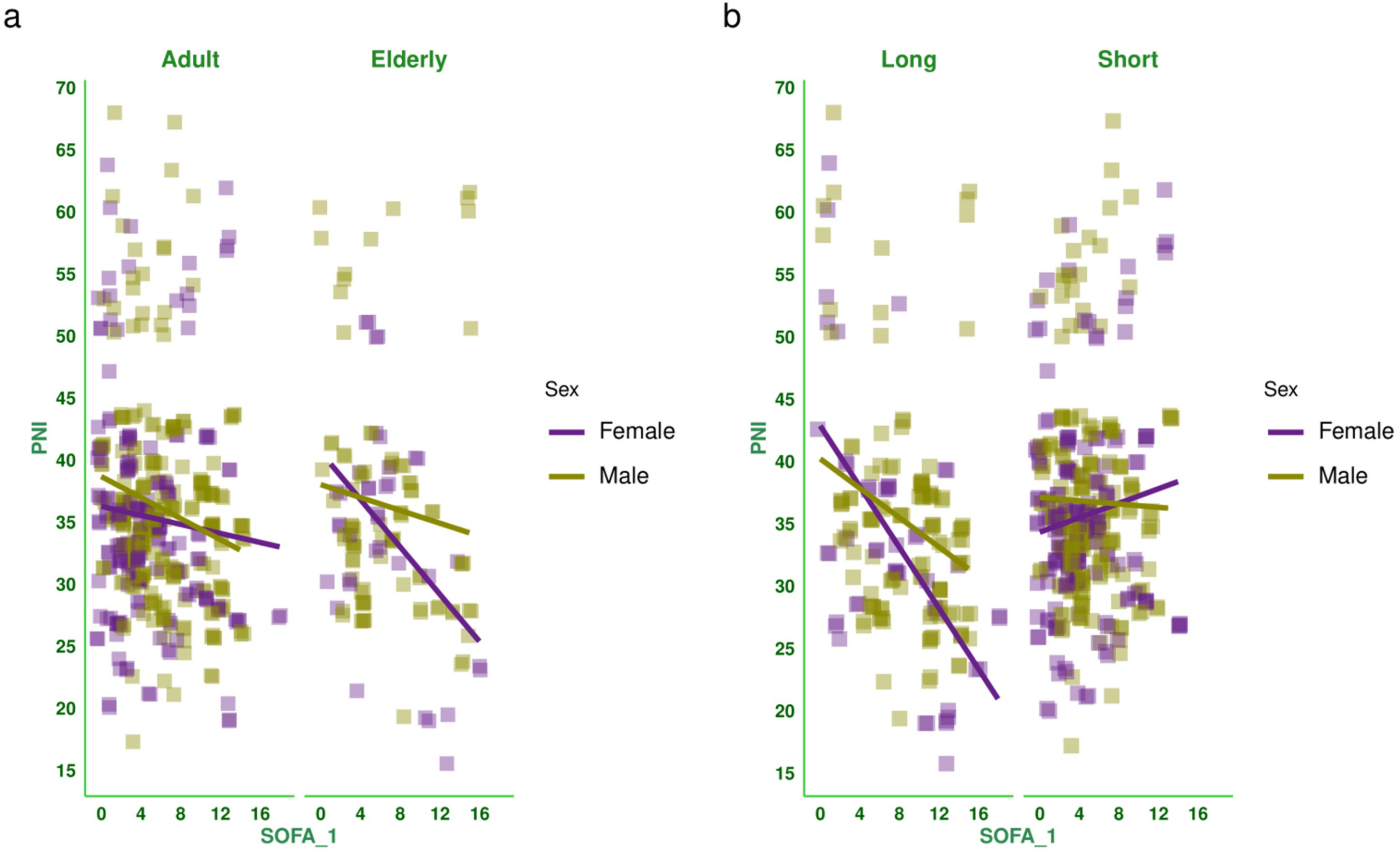
Correlation between PNI and SOFA score based on the age group and duration of hospitalization The jitter plots show the correlation between baseline SOFA score and PNI of the study population. Panels a and b show the subgroup analyses by age group and hospitalization duration, respectively. The Spearman correlation was used to check the association. The SOFA scores were assessed on the day of admission, day 3, and day 7, as per the SOFA scoring system [14]. The statistical significance is set at p < 0.05. PNI: prognostic nutritional index, SOFA: sequential organ failure assessment score, SOFA_1: SOFA score at day 1

Figure 2 shows the correlation between baseline SOFA score and PNI of the subjects. There was a negative correlation between the parameters (-0.153, 95% CI: -0.233 to -0.071, p < 0.001). Figure 3 shows the subgroup analysis of this correlation between baseline SOFA score and PNI based on the outcome (i.e., death or discharge) and the difference in SOFA score at day 7 (<2 or ≥2). The majority of correlations were weakly positive. Figure 4 shows the subgroup analysis of the correlation between baseline SOFA score and PNI, stratified by vasopressor use (yes/no) and MODS (present/absent). There were weakly negative correlations between PNI and SOFA score [subjects on vasopressors: (-0.051, 95% CI: -0.163 to 0.061, p = 0.370), subjects not on vasopressors: (-0.202, 95% CI: -0.318 to -0.080, p = 0.001), subjects with MODS: (-0.069, 95% CI: -0.159 to 0.023, p = 0.143), and subjects without MODS: (-0.218, 95% CI: -0.399 to -0.020, p = 0.031)]. Figure 5 shows the subgroup analysis of the correlation between baseline SOFA score and PNI by age group (≤65 or >65 years) and hospitalization duration (>14 or ≤14 days). There were negative correlations between PNI and SOFA score [adult subjects (i.e., ≤65 years): (-0.136, 95% CI: -0.228 to -0.043, p = 0.005), elderly subjects (i.e., >65 years): (-0.203, 95% CI: -0.365 to -0.028, p = 0.023), subjects with long hospital stay (i.e., >14 days): (-0.371, 95% CI: -0.493 to -0.236, p < 0.001), and subjects with short hospital stay (i.e., ≤14 days): (0.050, 95% CI: -0.051 to 0.149, p = 0.332)]. Table 2 presents all correlation coefficients, their 95% CIs, and p-values.

**Table 2:** Correlation between PNI and SOFA score of the study population The Spearman correlation was used to test the association. The SOFA scores were assessed on the day of admission, day 3, and day 7, as per the SOFA scoring system [14]. The statistical significance is set at p < 0.05. PNI: prognostic nutritional index, SOFA: sequential organ failure assessment score, MODS: multiorgan dysfunction syndrome.

Parameters	Total (n = 557)		Male (n = 335)		Female (n = 222)	
	r (95% CI)	p-value	r (95% CI)	p-value	r (95% CI)	p-value
Total	-0.153 (-0.233 to -0.071)	< 0.001	-0.169 (-0.271 to -0.063)	0.002	-0.147 (-0.273 to -0.015)	0.029
SOFA_diff <2 and death	0.439 (-0.668 to 0.134)	0.007	-0.132 (-0.553 to 0.343)	0.591	-0.623 (-0.844 to -0.220)	0.006
SOFA_diff ≥2 and death	-0.111 (-0.297 to 0.084)	0.263	-0.196 (-0.430 to 0.064)	0.137	-0.020 (-0.312 to 0.275)	0.894
SOFA_diff <2 and discharge	0.073 (-0.033 to 0.178)	0.176	0.025 (-0.111 to 0.160)	0.720	0.152 (-0.019 to 0.314)	0.081
SOFA_diff ≥2 and discharge	0.133 (-0.100 to 0.353)	0.261	0.148 (-0.145 to 0.417)	0.321	-0.053 (-0.431 to 0.341)	0.797
Vasopressor	-0.051 (-0.163 to 0.061)	0.370	-0.129 (-0.271 to 0.019)	0.088	0.001 (-0.172 to 0.173)	0.995
No vasopressor	-0.202 (-0.318 to -0.080)	0.001	-0.237 (-0.380 to -0.084)	0.003	-0.112 (-0.309 to 0.094)	0.286
MODS	-0.069 (-0.159 to 0.023)	0.143	-0.089 (-0.203 to 0.027)	0.133	-0.049 (-0.196 to 0.100)	0.520
No MODS	-0.218 (-0.399 to -0.020)	0.031	0.357 (-0.578 to -0.087)	0.011	-0.111 (-0.383 to 0.179)	0.454
Adult	-0.136 (-0.228 to -0.043)	0.005	-0.195 (-0.312 to -0.072)	0.002	-0.084 (-0.226 to 0.061)	0.255
Elderly	-0.203 (-0.365 to -0.028)	0.023	-0.120 (-0.321 to 0.092)	0.266	-0.431 (-0.662 to -0.124)	0.008
Long duration	-0.371 (-0.493 to -0.236)	< 0.001	-0.269 (-0.424 to -0.098)	0.002	-0.583 (-0.741 to -0.364)	< 0.001
Short duration	0.050 (-0.051 to 0.149)	0.332	-0.024 (-0.159 to 0.112)	0.728	0.124 (-0.026 to 0.269)	0.106

## Discussion

In this cross-sectional study, 557 patients were assessed. Their median age was 59.0 (52.0-65.0) years. There were 335 (60.14%) male subjects. The average hospital stay was 12.0 (9.0-16.0) days. 175 (31.42%) subjects were hospitalized for > 14 days. 459 (82.41%) subjects had MODS. Vasopressors were required for 306 (54.94%) patients. The median SOFA score during admission was 5.0 (3.0-8.0). There was a negative correlation between SOFA score at day 1 and PNI (-0.153, 95% CI: -0.233 to -0.071, p < 0.001). Female and male subjects demonstrated similar negative correlations. The subgroup analyses also showed similar findings. Our observations regarding the PNI matched those of Xie et al. [18] and Huang et al. [22].

The SOFA score is globally used to monitor the prognosis and morbidity of critically ill patients. However, its calculation warrants time and multiple factors [14,17]. Newer biomarkers for sepsis include CRP, IL-6, procalcitonin, ICAM-1, and adrenomedullin [15,16]. These biomarkers possess less practicality due to their higher cost and limited availability [15,17]. In our study, we have analyzed both SOFA scores and PNI of the study subjects. Our findings suggest that these two values have an inverse relation. The studies by Xie et al. [18] and Huang et al. [22] supported our study findings.

Systemic immune-inflammation index (SII) is another indicator of inflammatory, immune-mediated diseases, sepsis, and septic shock [26]. Nowadays, the PNI has emerged as an efficient composite biomarker for sepsis, encompassing nutritional aspects alongside immune and inflammatory responses [18-21]. Lower serum albumin levels are generally observed in malnourished individuals [18]. Our body’s immune function is positively correlated with the lymphocyte counts [26]. Patient prognosis is positively related to nutritional status. In patients with sepsis, inadequate nutrition results in worse survival rates [27]. PNI is a useful tool for determining the connection between a patient's prognosis and immune-nutritional status [27,28]. As PNI uses only serum albumin and absolute lymphocyte count for its calculation, it can be employed by healthcare providers at even the primary care level.

Strengths and limitations

The strength of the study is the evaluation and correlation of PNI and SOFA score among the study subjects. Our study also had a few limitations. First, a single-centred study and a small sample size limit the generalizability of the findings. Second, missing data on hemodynamic, renal, hepatic, and glycemic parameters, antibiotics used, concomitant medications, comorbidities, daily fluid intake, and urine output led us to exclude some patients. Third, referred patients were excluded because their indices could have been miscalculated owing to previous interventions. Fourth, the complete data for the patients who died within or stayed for less than seven days were not available and were not analyzed in this study.

## Conclusions

The study population had lower serum albumin and absolute lymphocyte values. Male and female participants showed similar serum albumin levels, lymphocyte counts, PNI, and SOFA scores. The correlation between the PNI and the SOFA score was weakly negative. The subgroup analyses showed similar observations. Our study findings cannot be generalized due to a single-centre study design, a small sample size, and missing data on renal, hepatic, glycemic, and hemodynamic parameters, antibiotics used, daily fluid intake, urine output, concomitant medications, and comorbidities. We suggest prospective, multicenter studies with larger sample sizes and longer follow-up to explore the potential of PNI as a biomarker for sepsis.
